# Anti-Asian racism related stigma, racial discrimination, and protective factors against stigma: a repeated cross-sectional survey among university students during the COVID-19 pandemic

**DOI:** 10.3389/fpubh.2023.958932

**Published:** 2023-09-13

**Authors:** Bernadette Boden-Albala, Xueting Ding, Nessa Ryan, Sara Goodman, Jeffrey Wing, Miryha Gould Runnerstrom, Desiree Gutierrez, Brooke Gibbs, John Michael Robb, Emily Drum

**Affiliations:** ^1^Program in Public Health, Susan and Henry Samueli College of Health Sciences, University of California, Irvine, Irvine, CA, United States; ^2^Department of Health, Society and Behavior, Program in Public Health, Susan and Henry Samueli College of Health Sciences, University of California, Irvine, Irvine, CA, United States; ^3^Department of Epidemiology and Biostatistics, Program in Public Health, Susan and Henry Samueli College of Health Sciences, University of California, Irvine, Irvine, CA, United States; ^4^Department of Neurology, School of Medicine, Susan and Henry Samueli College of Health Sciences, University of California, Irvine, Irvine, CA, United States; ^5^School of Medicine, Department of Pediatrics-Infectious Diseases, Stanford University, Stanford, CA, United States; ^6^College of Public Health, The Ohio State University, Columbus, OH, United States; ^7^School of Nursing and Health Studies, University of Washington Bothell, Bothell, WA, United States

**Keywords:** COVID-19, anti-Asian racism, anti-AAPI racism, stigma, mental health, college students, COVID-19 stigma among Asian students

## Abstract

**Background:**

Since the onset of the COVID-19 pandemic in March 2020, reports of anti-Asian American or Pacific Islander (AAPI) hate have increased in the United States. Institutions of higher education provide a unique opportunity to examine COVID-19 related stigma and protective factors in AAPI young adults enrolled in college.

**Objective:**

The goal of this research was to examine COVID-19 related stigma among a diverse college student population. We posited that AAPI students experience more racial discrimination, internalized stigma, and/or anticipated racial discrimination than other students. We also sought to identify protective behavioral factors against stigma.

**Methods:**

This study includes data from a repeated cross-sectional survey that was administered among college students at a large public university in the United States in April (*n* = 1,359) and November 2020 (*n* = 1,196). All university enrolled students with an active email account were eligible to participate in the online survey, which included questions about COVID-19 stigma (anticipated, enacted, internalized), stigma resistance, sources of COVID-19 information, lifestyle behaviors, and sociodemographic information. Binary logistic regression models were utilized to assess differences in stigma between race and ethnic groups and to identify factors associated with stigma.

**Results:**

AAPI students were more likely to experience all three types of stigma compared to other race and ethnic groups. AAPI students in both waves were at least 2 times more likely to experience enacted stigma and 7.3 times more likely to experience anticipated stigma in the earlier wave compared to non-Hispanic White students. Students who had experienced enacted stigma were more likely to experience anticipated stigma, and those who experienced enacted and anticipated stigma were more likely to experience internalized stigma. Higher education level, living with neighbors/roommates, maintaining a healthy lifestyle, and thinking positively about oneself may act as protective factors against different types of stigma.

**Conclusion:**

AAPI students have a greater risk of experiencing COVID-19 stigma compared to those from other race and ethnic groups. Universities should combat anti-AAPI sentiments and COVID-19 stigma and promote public health efforts to build resistance against the negative effects of stigma.

## Introduction

Since the onset of the COVID-19 pandemic in March 2020, reports of anti-Asian American or Pacific Islander (AAPI) hate have increased in the United States (U.S.) According to the national coalition Stop AAPI Hate, 11,500 anti-AAPI hate incidents were reported across the U.S. between March 2020 and March 2022 (1). These incidents included hateful tirades, online abuse, refusal of services, shunning, physical assault, property damage, and robbery, with 67% of reported incidents involving harassment such as verbal or written hate speech or inappropriate gestures (1). In the COVID-19 Effects on the Mental and Physical Health of AAPI Survey Study (COMPASS), 60% of respondents reported experiencing discrimination within the first year of the pandemic (2), and 74% agreed with at least one COVID-19 related racial bias belief (3). Discrimination is closely linked with stigma, as the interplay between harmful stereotypes and structural power dynamics can lead to the othering of individuals or entire groups of people (4). As with other previous infectious disease outbreaks (e.g., HIV, tuberculosis, Zika), stigma emerged during the COVID-19 pandemic (4).

COVID-19 stigma initially manifested towards individuals from Wuhan, China but was subsequently generalized in varying intensity to others, most specifically among those of AAPI descent (5, 6). Initial COVID-19 stigmatization of AAPI individuals was often exacerbated by misinformation and anti-AAPI sentiments perpetuated by the media and government leadership (7). Individuals reporting discrimination in COMPASS included those of Hmong, Chinese, Korean, Filipino, Japanese, Vietnamese, Asian, Indian, and Native Hawaiian and Pacific Islander descent (2). In another national survey assessing self-reported COVID-19 related racial and ethnic discrimination, Chinese, Korean, Japanese, Vietnamese, and other AAPI individuals were almost four times more likely to experience COVID-19 related discrimination compared to White individuals (8).

Stigma is a persistent and critical public health issue that stems from a lack of understanding, misleading or inaccurate information, and stereotypes (9). The concept of stigma has evolved over time and operates on the individual, interpersonal, and population levels (9). At the individual level, stigma can be internalized if an individual agrees with others’ negative beliefs related to their identity or participation within a group perceived to be at greater risk of transmitting COVID-19. Internalized stigma can lead to greater emotional, mental, and physical health consequences including shame, lower self-esteem, fear, anxiety, depression, and suicidal ideation (10, 11). Stigma may also be anticipated, meaning an individual fears future stigmatization or discrimination because of this attribute, which can also have a negative effect on wellbeing (12). Additionally, anticipated stigma can occur whether or not one is actually exposed to stigma (12, 13). Although stigma can lead to negative mental, emotional, and physical outcomes, one may be exposed to stigma but avoid the associated negative outcomes due to protective factors that increase stigma resistance. Stigma resistance is the ability to use one’s own knowledge, experiences, and skills to fight stigma at the personal, peer, or public level (14–16).

A growing body of research focuses on examining the experiences of the AAPI community during the COVID-19 pandemic and the negative effect that COVID-19 related racial discrimination and stigma have had on mental health and wellbeing (2, 17, 18). One early study on self-reported racial discrimination among AAPI individuals found a 30% increase in reported discrimination around the pandemic, with 40% of respondents reporting an increase in anxiety, depressive symptoms, and sleep difficulties (19). In a survey of Chinese families living in the U.S., symptoms of anxiety and poorer psychological well-being were associated with experiences of racial discrimination related to COVID-19 among both parents and their children (20).

The young adult AAPI community may be particularly vulnerable to the deleterious effects of COVID-19 on mental health and well-being. Among young adults identifying as racial and ethnic minorities, COVID-19-related stigma could be an under-examined predictor of increasing rates of anxiety and depression (21). In the U.S., recent levels of anxiety and depression have risen with 42% of young adults aged 18–29 reporting anxiety and 36% reporting depression (22). Levels of suicidal ideation in young adults are similarly rising, notably more prevalent among males than females, as are reports of substance use, which is often used to cope with seemingly overwhelming stressors (22). Among the AAPI population, suicide rates were the highest in adolescents and young adults ages 15–24 and 25–34 (23). AAPI individuals are also less likely to seek mental health services due to lack of access to care, perceived need, and mental health stigma (24–26). Using data from the California Health Interview Survey (CHIS), researchers found that, among AAPI individuals, experiencing or witnessing COVID-19 related hate caused serious psychological distress and that these experiences were concentrated in the younger population (27).

Of the publications that specifically examine the experiences of COVID-19 stigma among AAPI populations, few examine the protective factors against stigma among young AAPI adults or college students in the U.S. One study found that those with lower educational levels are more likely to experience infectious disease related to enacted or perceived public stigma, including COVID-19 related stigma. This association may be due to the relationship between education level and the ability to identify misinformation (28). Another study identified social support as a critical factor associated with increased resilience to stigma (19).

Institutions of higher education provide a unique opportunity to examine COVID-19 related stigma in young adults enrolled in college. While data exist suggesting AAPI populations may experience increased COVID-19 stigma, less is known about the experience of the AAPI student population as well as the experience of other college groups related to COVID-19 stigma. Given the significant race and ethnic disparities that emerged early in the pandemic, it is possible that other student populations may have experienced COVID-19 related stigma as well. Finally, a scarcity of data exists around factors associated with COVID-19 resilience. The goal of this research was to examine COVID-19 related stigma among a diverse college student population. We posit that AAPI students experience more racial discrimination (enacted stigma), internalized stigma, and/or anticipated racial discrimination (anticipated stigma) compared to other students. We also sought to identify behavioral factors associated with anticipated stigma and to identify which factors act as protective mechanisms against internalized stigma related to COVID-19.

## Materials and methods

### Setting and population

This study was conducted at a large public research university located on the West Coast of the U.S. This university is a Minority Serving Institution, designated as both an Asian American and Native American Pacific Islander-Serving Institution and a Hispanic-Serving Institution. In fall 2019, the university’s population comprised 36,303 students who identified as AAPI (36%), Hispanic (22%), international (19%), White (16%), African American (3%), and unknown/did not identify (3%). Among those who reported being international students, the top sending countries included China (74%), India (6%), and South Korea (4%). Approximately 79% of those enrolled were undergraduates, and almost half of those undergraduates (47%) were first generation college students (29).

### Data collection

**Figure 1:**
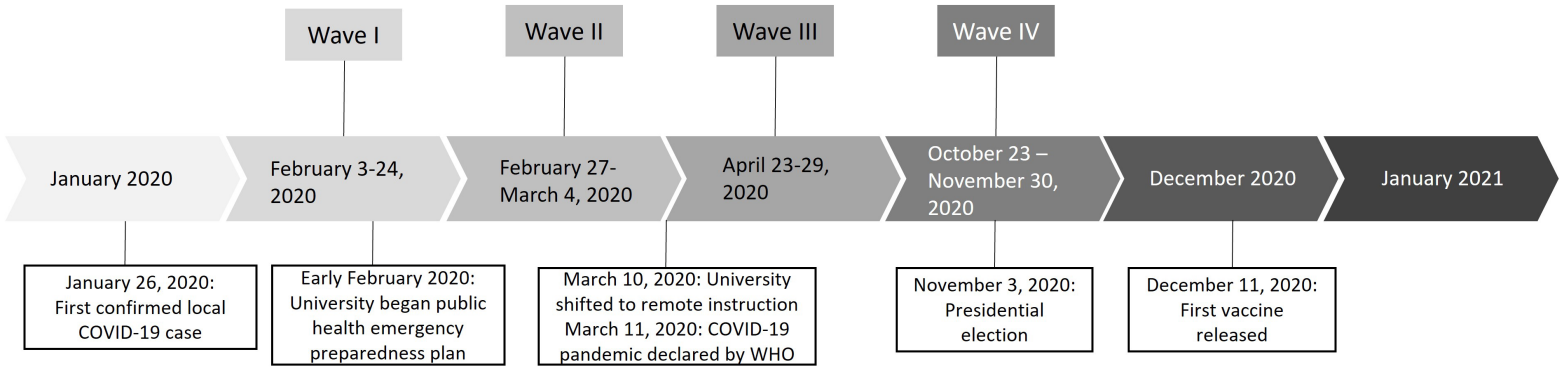
COVID-19 student survey timeline.

In late January 2020, university leadership began to make plans in preparation for COVID-19 on campus. To better understand health behaviors, we developed a campus wide survey to gauge students’ concerns regarding the COVD-19 virus, understanding of disease transmission, use of protective health behaviors, and sources of COVID-19 information. In a pilot test with students, the survey took approximately 10–15 min to complete. We administered an anonymous, repeated cross-sectional survey, with a total of four survey waves. (See Figure 1 for timeline). We initiated the Wave I survey in February 2020. Starting with the Wave III survey in April 2020, we enhanced the survey questions and added questions about mental health, healthy coping mechanisms, and COVID-19 stigma. We administered the Wave IV survey in November 2020. Thus, we include only results from Wave III and IV surveys in these analyses related to understanding COVID-19 related stigma among a college student population. This study was certified as exempt (Category 2) from ethical approval using a self-determination form provided by the university’s Institutional Review Board (IRB).

### Recruitment and sample

All university enrolled students with an active email account during the study period were eligible and invited to participate in the online survey via their university email. Those who completed the survey were entered in a raffle to win a $50 gift card for a food delivery app. Approximately 31,000 surveys were sent out in each of Waves III and IV, and 2,272 and 1,611 students completed the surveys, respectively. However, 913 responses from Wave III and 415 responses from Wave IV were excluded from analysis due to incomplete submissions.

### Outcome variables

This study has three key stigma outcome variables: enacted stigma (experience of overt discrimination), anticipated stigma (fear of future stigmatization), and internalized stigma (agreement with others’ negative beliefs about one’s identity). We adapted items from the HIV/AIDS Stigma Instrument for People Living with AIDS (HASI-P) (30) for the context of COVID-19 to measure these different types of stigma. HASI-P was previously adapted by the authors (31) and is often adapted for various stigmatized health conditions (32, 33). For *enacted stigma*, we asked respondents to report how often they had experienced being mocked, having friendships dissolve, feeling verbally abused, or being avoided because of others’ perceptions of the respondent’s racial identity or perceived racial identity and their risk of transmitting COVID-19 to others within the past month. For *anticipated stigma*, we asked respondents to report how often they believed they would be mocked, have friendships dissolve, experience verbal abuse, or be avoided because of others’ perceptions of the respondent’s racial identity or perceived racial identity and their risk of transmitting COVID-19 to others within the next month. For *internalized stigma*, we asked respondents to report if they ever felt ashamed or worthless because of others’ perceptions of their racial identity and their risk of transmitting COVID-19 to others. Response options for enacted, anticipated, and internalized stigma questions included “most of the time,” “several times,” “once or twice,” and “never.”

Stigma resistance was measured based on the activity type: (1) protective health behaviors to resist stigma and (2) actions to resist/address stigma at the personal, peer, and public level. Students were asked how often they participated in protective health behaviors and how much they agreed with statements about resisting and addressing COVID-19 stigma; these items were adapted from the Stigma Resistance Scale (16). These survey items were divided into 3 sets of questions: protective behaviors, stigma resistance activities, and stigma addressing activities.

### Covariates

Socio-demographic characteristics include age (in years); gender (male/female/transgender female/transgender male/gender non-conforming/not listed/prefer not to answer); education (undergraduate/graduate); race (select all that apply: American Indian or Alaska Native/Asian/Black or African American/Native Hawaiian or Other Pacific Islander/White/Other); ethnicity (Hispanic or Latinx/not Hispanic or Latinx); occupation (full-time employed/part-time employed/unemployed); residence (urban/rural); and household membership (select all that apply: living alone/significant other/partner or spouse/parent(s)/your child or children/sibling(s) (brothers or sisters)/extended family/neighbor(s)/ friend(s)/roommate(s)—not family or friend). Gender was collapsed as a binary indicator for females and non-females. Although recent research shows that transgender and non-binary individuals are more susceptible to depression and other negative health outcomes (34, 35), our sample included few individuals who identified as non-binary or transgender. Therefore, these gender categories were too small for individual analyses. Race and ethnicity were collapsed into AAPI (Asian and Non-Hispanic Pacific Islanders), Hispanic or Latino, Non-Hispanic White, and Other Non-Hispanic (Non-Hispanic Black and Non-Hispanic American Indian). Any student who identified as “Asian” was categorized as AAPI, as we were missing data on AAPI subcategories. Non-Hispanic Black and Non-Hispanic American Indian was collapsed into Other Non-Hispanic due to the small number of observations. Living situation was categorized as living alone, living with family, living with friends, and living with neighbors/roommates. Employment was collapsed into two categories: not employed or employed (part-time or full-time). Students were asked about their sources of COVID-19 information which included news media, social media (university or non-university), podcasts (university or non-university), government websites, university websites, friends and family, and emails from the university (36).

### Statistical analysis

Descriptive statistics were calculated to summarize the survey respondents by waves and demographic variables. We assessed the impact of missing data using the chi-square test of homogeneity to examine if the distribution of observations with missing race and ethnicity was significantly different from observations with race and ethnicity across different demographic and outcome variables (Supplementary Table S1). Those with missing race and ethnicity observations were categorized as “Missing” for their race and ethnicity category to maintain their inclusion in the analyses.

Racial differences in stigma were estimated using binary logistic regression models comparing any frequency of stigma to no stigma. Odds of enacted, anticipated, and internalized stigma were separately estimated in unadjusted models as no confounders were identified in our Directed Acyclic Graphs (DAGs) (37) based on existing literature for each type of stigma (Supplementary Figures S2–S4). Separate DAGs were used to identify confounders of anticipated stigma (Supplementary Figure S5) and internalized stigma (Supplementary Figure S6). Supplementary Figure S5 illustrates how demographic, socio-economic, social environmental factors, media consumption, stigma resistance, and protective health behaviors are associated with anticipated stigma, and we then tested for multicollinearity. Final model covariates for the odds of anticipated stigma were enacted stigma, age, gender, race and ethnicity, education, employment, rural/urban status, the people participants lived with, media source of COVID-19 information, daily protective health behaviors for stigma, stigma addressing activities, and stigma resistance activities.

Supplementary Figure S6 includes demographic, socio-economic, social environmental factors and media use, stigma resistance, and protective health behaviors as confounders of the race and ethnicity internalized stigma association. It is worth noting that anticipated stigma is included as a covariate, since anticipated stigma can lead to internalized stigma (11, 12).

All analyses were run via SAS 9.4 (SAS Institute, Cary NC.) All regression results are presented as odds ratios and their corresponding 95% confidence intervals (CI).

## Results

### Descriptive statistics

A total of 1,359 and 1,196 student responses were included in Waves III and IV, respectively. The mean age of participants in Wave III was 22.59 years old (SD = 5.28; median age of 21 years), and the mean age of participants in Wave IV was 22.18 years (SD = 5.00; median age of 21 years) (Table 1). Most participants were female (Wave III: 68.73%, Wave IV: 69.98%), undergraduate (Wave III: 74.17%, Wave IV: 69.73%), and were not employed (Wave III: 59.90%, Wave IV: 55.94%). The majority of students identified as AAPI (Wave III: 39.00%, Wave IV: 45.40%), Hispanic or Latino (Wave III: 21.34%, Wave IV: 21.24%), or non-Hispanic White (NHW) (Wave III: 20.60%, Wave IV: 25.17%). A smaller proportion of students identified as “Other Non-Hispanic” (Wave III: 2.58%, Wave IV: 2.68%) (Table 1). These self-identified race and ethnic distributions are consistent with the university’s current enrollment demographics. Race and ethnicity data were missing for 16.48% of Wave III and 5.52% of Wave IV.

**Table 1:** Descriptive summary by waves.

Variables	Wave III (*n* = 1,359)		Wave IV (*n* = 1,196)	
Race	*N*	%	*N*	%
Asian and Pacific Islander (AAPI)	530	(39.00%)	543	(45.40%)
Hispanic or Latinx	290	(21.34%)	254	(21.24%)
Other Non-Hispanic	35	(2.58%)	32	(2.68%)
Non-Hispanic White	280	(20.60%)	301	(25.17%)
Missing	224	(16.48%)	66	(5.52%)
Gender				
Male	425	(31.27%)	359	(30.02%)
Female	934	(68.73%)	837	(69.98%)
Education				
Undergraduate	1,008	(74.17%)	834	(69.73%)
Graduate	344	(25.31%)	360	(30.10%)
Missing	7	(0.52%)	2	(0.17%)
Employment status				
Employed	545	(40.10%)	527	(44.06%)
Unemployed	814	(59.90%)	669	(55.94%)
Age				
Mean	22.59		22.18	
Median	21.00		21.00	
Standard deviation	5.28		5.00	
Outcome variables				
Enacted stigma	169	(12.44%)	121	(10.12%)
Anticipated stigma	380	(27.96%)	194	(16.22%)
Internalized stigma	197	(14.50%)	153	(12.79%)

### Binary logistic regression results

Overall, the prevalence of any type of stigma was observed in 493 out of 1,359 (36.28%) participants in Wave III and 284 out of 1,196 (23.75%) participants in Wave IV, both representing a significant percentage of respondents. In terms of stigma subtypes, the prevalence of enacted stigma was 12.44% in Wave III (*n* = 169) and 10.12% in Wave IV (*n* = 121). Anticipated stigma was reported by 27.96% (*n* = 380) of participants in Wave III and 16.22% (*n* = 194) of participants in Wave IV. Internalized stigma was reported by 14.50% and 12.79% of the participants in Waves III and IV, respectively.

AAPI respondents reported all three types of stigma significantly more than other race and ethnic groups for both waves; however, this trend decreased from Wave III to Wave IV (Table 2). In Model 1, AAPI students were over 2 times more likely to experience enacted stigma compared to NHW [Wave III: OR 2.6 (95% CI: 1.56–4.31); Wave IV: OR 2.1 (95% CI: 1.23–2.55)]. Similarly, AAPI students were also 7.3 times more likely to experience anticipated stigma compared to NHW (95% CI: 4.72–11.35) in Wave III and 4.1 times more likely (95% CI: 2.53–6.68) in Wave IV. Other Non-Hispanic participants also had significantly higher odds of experiencing anticipated stigma compared to NHW in both Waves (Wave III: aOR = 3.4; 95% CI: 1.43–7.98); Wave IV: aOR = 4.4; 95% CI: 1.78–11.09). Additionally, AAPI students were the only ones to experience more internalized stigma compared to NHW in both Wave III (aOR = 1.62; 95% CI: 1.05–2.51) and Wave IV (aOR = 1.58; 95% CI: 1.02–2.47).

**Table 2:** Binary logistic regression results of race and ethnic differences in enacted, anticipated, and internalized stigma by survey wave.

	Wave III		Wave IV	
Variables	aOR	95% CI	aOR	95% CI
Outcome: enacted stigma				
Race				
Asian and Pacific Islander (AAPI)	2.59**	(1.56, 4.31)	2.09**	(1.23, 2.55)
Hispanic or Latinx	0.86	(0.45, 1.66)	1.77	(0.96, 3.26)
Other Non-Hispanic	2.69	(1.00, 7.24)	2.12	(0.67, 6.67)
Missing	2.57**	(1.45, 4.57)	0.96	(0.32, 2.91)
Non-Hispanic White	Reference	Reference	Reference	Reference
Outcome: anticipated stigma				
Race				
Asian and Pacific Islander (AAPI)	7.32**	(4.72, 11.35)	4.11**	(2.53, 6.68)
Hispanic or Latinx	0.96	(0.54, 1.71)	1.72	(0.95, 3.09)
Other Non-Hispanic	3.38**	(1.43, 7.98)	4.44**	(1.78, 11.09)
Missing	6.81**	(4.19, 11.04)	1.84	(0.78, 4.35)
Non-Hispanic White	Reference	Reference	Reference	Reference
Outcome: internalized stigma				
Race				
Asian and Pacific Islander (AAPI)	1.62*	(1.05, 2.51)	1.58*	(1.02, 2.47)
Hispanic or Latinx	1.21	(0.73, 2.01)	1.16	(0.68, 1.99)
Other Non-Hispanic	1.34	(0.48, 3.70)	1.67	(0.60, 4.67)
Missing	1.44	(0.85, 2.42)	1.25	(0.54, 2.86)
Non-Hispanic White	Reference	Reference	Reference	Reference

*p < 0.05, **p < 0.01; any student who included “Asian” as one of their races was categorized as AAPI; Other Non-Hispanic includes Non-Hispanic Black and Non-Hispanic American Indian; Non-Hispanic White is the reference group; Stigma indicators were coded as binary variables (1 = participants selected most of the time, several times, or once/twice; 0 = never).

After adjusting for demographic (age and gender), social (education, residence, and employment), environmental (living situation), and behavioral factors (source of COVID-19 information; protective health behaviors to resist stigma; and actions to resist/address stigma at the personal, peer, and public level), race and ethnicity remained significantly associated with anticipated stigma (Table 3). In models 4 and 5, all covariates remained as there was no multicollinearity among variables (Supplementary Table S2). These adjusted models also demonstrate that students who experienced enacted stigma were 40–50 times more likely (Wave III, 95% CI: 18.05–82.79; Wave IV, 95% CI: 24.21–111.18) to experience anticipated stigma compared with those who did not experience enacted stigma. Students who reported agreement with the statement “Resisting stigma means speaking up when others say negative things about Asians or Asian Americans regarding COVID-19” were more likely to experience anticipated stigma (aOR = 2.38; 95% CI: 1.12–5.03) in Wave III.

**Table 3:** Logistic regression results of sociodemographic, social environmental, and behavioral factors associated with anticipated stigma by wave.

	Wave III		Wave IV	
Variables	aOR	95% CI	aOR	95% CI
Enacted stigma	38.66**	(18.05, 82.79)	51.88**	(24.21, 111.18)
Race: Asian and Pacific Islander (AAPI)	7.50**	(3.72, 15.15)	7.06**	(2.79, 17.91)
Race: Non-Hispanic Others	2.58	(0.58, 11.48)	12.98*	(2.43, 69.39)
Race: missing	7.68**	(3.55, 16.63)	1.48	(0.27, 8.17)
Race: Non-Hispanic White	Reference	Reference	Reference	Reference
Limiting your news consumption to sources considered reliable (meaning with accurate and timely public health information regarding COVID-19)	1.00	(0.53, 1.88)	3.59*	(1.09, 11.83)
Maintaining a healthy lifestyle: getting enough sleep, eating well, exercising, avoiding excessive alcohol or drugs	0.92	(0.49, 1.70)	0.27*	(0.13, 0.59)
Resisting stigma means speaking up when others say negative things about AAPI regarding COVID-19	2.38*	(1.12, 5.03)	0.51	(0.20, 1.31)

*p < 0.05, **p < 0.01; only significant variables are shown on this table; the full table is shown on Supplementary Table S3.

Race and ethnicity were not associated with internalized stigma in the adjusted Model 5 (Table 4). However both enacted stigma [Wave III (aOR = 3.39; 95% CI: 1.87–6.14); Wave IV (aOR = 4.98; 95% CI: 2.44–10.19)] and anticipated stigma [Wave III (aOR = 2.72; 95% CI: 1.59–4.64); Wave IV (aOR = 3.33; 95% CI: 1.72–6.47)] were significantly associated with higher odds of experiencing internalized stigma when controlling for the other covariates. These associations were of higher magnitude in Wave IV.

**Table 4:** Logistic regression results of demographic, social, environmental, and behavioral factors associated with internalized stigma by wave.

	Wave III		Wave IV	
Variables	aOR	95% CI	aOR	95% CI
Enacted stigma	3.39**	(1.87, 6.14)	4.98**	(2.44, 10.19)
Anticipated stigma	2.72**	(1.59, 4.64)	3.33**	(1.72, 6.47)
Graduate student	0.37*	(0.15, 0.91)	0.76	(0.30, 1.93)
Live with roommates or neighbors	0.76	(0.29, 2.01)	0.25*	(0.07, 0.83)
Live with friends	2.96*	(1.10, 7.97)	0.60	(0.18, 1.96)
Using telehealth options (phone-based or online) for therapy	1.14	(0.64, 2.05)	1.93*	(1.05, 3.55)
To resist stigma, I think positive things about myself	0.64	(0.39, 1.06)	0.48*	(0.27, 0.85)

*p < 0.05, **p < 0.01; only significant variables are shown on this table; the full table is shown on Supplementary Table S4.

A number of protective factors were associated with decreased internalized stigma including graduate education level, living with roommates or neighbors in university mandated “pod” cohorts (i.e., groups of students who lived, ate, and socialized exclusively with each other), and positive self-thought. Compared to undergraduate students, being a graduate student was associated with a lower risk of internalized stigma in Wave III (aOR = 0.37; 95% CI: 0.15–0.91) but not in Wave IV. In both waves, participants living within university mandated “pod” cohorts were less likely to experience internalized stigma compared to those who did not, and this finding was significant in Wave IV (aOR = 0.25; 95% CI: 0.07–0.83). However, those who lived with friends in Wave III (aOR = 2.96; 95% CI: 1.10–7.97) were more likely to experience internalized stigma. Students who reported agreement with the statement “To resist stigma, I think positive things about myself” were two times less likely to experience internalized stigma compared to those who did not (aOR = 0.48; 95% CI:0.27–0.85).

Behaviors associated with less anticipated stigma included maintaining a healthy lifestyle (aOR = 0.27; 95% CI: 0.13–0.59).

See Supplementary Tables S3, S4 for further results.

## Discussion

This study is one of the first to describe COVID-19 stigma and stigma subtypes among young adult students in a university setting with an emphasis on understanding stigma through the lens of AAPI students. While COVID-19 stigma was experienced broadly across the student population, enacted, anticipated and internalized stigma were significantly greater among students identifying as AAPI. This study contributes meaningfully to a growing body of research on COVID-19 stigma and the AAPI experience; adds to the literature on young AAPI adults; and includes new knowledge on the identification of protective factors against COVID-19 stigma.

During the first year of the pandemic, U.S. leadership evoked anti-AAPI sentiments through comments that associated both place and origin of COVID-19 outbreaks with the AAPI population, resulting in stigma and increased reports of anti-AAPI racial discrimination. In a survey of U.S. residents during the pandemic, 40% of respondents reported that they would engage in at least one discriminatory behavior toward a person of AAPI descent, and discrimination towards AAPI individuals was also associated with being fearful of COVID-19 and having less accurate knowledge of the virus (38). Our work demonstrates the significant impact of anti-AAPI sentiment on several communities of students but most meaningfully on AAPI students across university settings even in federally designated AAPI-serving institutions of higher education.

Our results reveal that the experience of COVID-19 stigma among AAPI students was consistent across two time points: the early acute pandemic experience in March 2020 and a more sub-acute pandemic experience in November 2020. The early COVID-19 experience can be characterized by an acute population-level state of fear of an unknown virus. During this time, university students transitioned to remote instruction as mandatory stay-at-home orders were put into effect statewide. By November 2020, our university campus, like many others, employed a hybrid education model, and students were more likely to return to living in residence halls or with friends. Despite the increase in scientific knowledge around COVID-19, including significant treatment advances and anticipated vaccines, during the time interval between the two waves, the consistent experience of stigma among AAPI students highlights a bias toward AAPI populations that did not significantly diminish.

A few studies reported that AAPI subgroups may experience more or different stigma than others (1, 8). Our study examined COVID-19 stigma among AAPI students as an aggregate. We were unable to conduct analyses by subgroup because we only captured AAPI student self-identified subgroups in Wave IV and the latter part of Wave III. In the sample that did include AAPI subcategories, the cell numbers for some subgroups were too small to conduct meaningful analyses. In Wave IV, we did find that any AAPI subgroup indicator was associated with enacted or internalized stigma. As for anticipated stigma, however, Filipino and Vietnamese participants were shown to have higher risk compared to other AAPI students (data not shown). This analysis is limited, however, and future studies should look at AAPI subgroups.

While this analysis focuses primarily on AAPI students, our convenience sample included students from other race and ethnic backgrounds. We observed that other self-identified non-Hispanic students also were more likely to experience anticipated stigma compared to NHW students. This collapsed category included American Indian, Alaska Native, Black and African American students. Students in these groups could experience stigma for a number of reasons. For example, early in the pandemic, Black communities were highlighted among those populations with highest rates of severe COVID-19 disease and death in the U.S., which may have enhanced fear and uncertainty and heightened feelings of vulnerability.

### Protective factors against stigma

Crucial to informing public health efforts to address stigma among young adults is the identification of associated factors and protective mechanisms which can increase stigma resistance. Our Wave III results indicate that graduate students were less likely to internalize stigma compared to undergraduate students. This finding is consistent with other studies that have reported higher education level as a protective factor against stigma (23–25). For example, older or graduate-level students may be less likely to experience personal stigma related to mental health compared to undergraduate students (39).

Across the different types of stigma, having access to one’s social network, such as living with others (40) and having adequate social support (41) can also help protect against the harmful effects of stigma. For instance, social support was found to significantly buffer the effect of COVID-19 related discrimination against depressive symptoms among AAPI individuals (19, 42). In Wave IV of our study, students who lived with neighbors or roommates were less likely to experience internalized stigma. A possible explanation for this finding could be that those who live with others have access to the protective factor of social support. Studies of other infectious and chronic illnesses have shown adequate social support to be a consistent protective factor against depression and poor quality of life due to internalized stigma (43–45). Future research should further examine the effect of adequate social support on COVID-19 stigma and mental health outcomes among young adults.

Our study also identified activities that were associated with a protective effect against stigma, including thinking positively about oneself (internalized stigma) and maintaining a healthy lifestyle (anticipated stigma). Stigma resistance is an ongoing process (46), and it may be useful for universities to promote behaviors that encourage students to maintain healthy lifestyle behaviors (e.g., eating well, exercising, getting enough sleep) and bolster self-esteem in addition to other COVID-19 risk reduction behaviors (e.g., handwashing, social distancing).

All of the protective factors identified in our results were found to be statistically significant in only one wave of data collection, either Wave III or Wave IV, which may have been impacted by students’ changing behaviors, social environments, and living arrangements as we moved from the early acute to subacute pandemic experience.

### Other factors

Our study also found that limiting news consumption to reliable sources was positively associated with experiencing anticipated stigma. According to Cultivation Theory, the public may develop ideas about society through their exposures to mass media, and studies have demonstrated that the media might operate as a route of stigma transmission around health-related topics (47, 48). News media resources may contribute to discriminatory beliefs and racial related stigma through selective exposure that leans towards certain beliefs or views (49). Further research should do more causal analyses regarding media exposure and COVID-related stigma to aid university administrators in making policy improvements.

Interestingly, living with friends, using telehealth options for therapy, and agreeing with the statement “Resisting stigma means speaking up when others say negative things about Asians or Asian Americans regarding COVID-19” were found to be associated with experiencing different types of stigma. Future research should conduct longitudinal analyses to disentangle the temporality of these associations.

## Limitations

Our study has some limitations. As the design is a repeated cross-sectional survey, it is not possible to comment on temporality or the causality of the relationship between the covariates. Future research should assess trends over time. Furthermore, this survey utilized a convenience sample and did not proportionately sample participants, which may decrease the generalizability. Students responded to the survey waves anonymously, and, given the rapid deployment of the survey as part of the university’s acute response to an unprecedented public health crisis on campus, it is possible there was some small overlap in respondents between Waves III and IV. However, we feel that the large sample size helps mitigate any impacts on our analyses.

The dataset features some missingness in the race and ethnicity data due to incomplete submissions. These omissions could be due to survey fatigue (50). Demographic questions were put at the end of the online survey to increase the response rate to questions about COVID-19 knowledge and attitudes at the beginning of the survey but may have led some participants to leave the demographic portion blank. Overall, Wave III was more likely to have missing race and ethnicity data. Between Waves III and IV, we improved the survey design to minimize missing data in subsequent waves by re-ordering the questions. The survey questions remained the same except for a few questions that were revised or added to Wave IV. For example, the race and ethnicity questions were improved to include AAPI subgroups. However, some literature suggests that AAPI and Hispanic respondents may be less likely to report race on surveys than NHW respondents (51). Further, the survey was administered during a time of heightened alert in which discussion of racism and anti-AAPI sentiment was prevalent in news cycles, and some participants may have intentionally not reported their race and ethnicity for this reason. It is possible that participants may have also intentionally skipped questions relating to stigma. Analysis of the missing data did not have a large impact on the results. Additionally, some measurement errors may exist in our findings. The items utilized to measure COVID-19 stigma resistance were based on existing literature and other existing, validated stigma resistance scales (16), however, this may be a limitation as the questions created were not validated.

## Conclusion

Institutions of higher education provide a unique opportunity to examine COVID-19 related stigma in young adults enrolled in college. The organized structure of these institutions also allows for targeted interventions to reduce stigma and enhance resilience. Despite the increased prevalence of COVID-19 stigma, there has been a lack of policies and communication set to protect vulnerable populations during the pandemic. Our results illustrate how young AAPI college students are at increased risk of experiencing stigma. To cultivate a safe campus that is stigma free, colleges should implement culturally targeted, anti-stigma COVID-19 interventions that are informed by anti-racism as well as by mental health stigma interventions that have been previously implemented among college populations (52). Possible interventions can include initiatives to increase awareness about stigma through social media campaigns, campus-wide communications, and events with interactive activities and giveaways to boost student engagement and participation in learning about stigma and how to combat it. Interventions should also leverage the campus’s existing resources such as student health centers and counseling centers to provide students with information and resources about COVID-19 and mental health. Additionally, universities should partner with their student organizations, advocacy groups, and cultural organizations to ensure the interventions are student-led and informed by student’s experiences, needs, concerns, and questions around COVID-19 stigma (52).

Overall, there has been no meaningful federal public health response (5). We call for nationwide interventions to build resiliency against stigma and mitigate the health and well-being consequences among individuals facing increased discrimination during the COVID-19 pandemic. Policies should be implemented to protect these individuals from further hate, harassment, and violence. In May 2021, Congress passed the federal COVID-19 Hate Crimes Act to begin to address the surge in hate crimes against the AAPI community during the COVID-19 pandemic (53). This act directs the Department of Justice to expedite reviews of hate crimes, creates a hate-crime reporting system, and requires that the Departments of Justice and Health and Human Services raise awareness of hate crimes during the COVID-19 pandemic (53). A need remains for policies aimed at prevention of hate crimes against vulnerable populations during the COVID-19 pandemic and beyond. Future research should assess trends in COVID-19 stigma throughout this dynamic pandemic. Recent media coverage has highlighted the significant disparities in COVID-19 transmission and severity of outcomes for African American, Hispanic, and Indigenous communities. Public health practitioners, researchers, and policy makers should be responsive to how AAPI and other historically marginalized populations share the stigma burden and plan policy, research, and programs accordingly to understand and address their needs. Importantly, as COVID-19 is likely to become an endemic disease, a long-term plan to build the capacity of individuals and communities to resist COVID-19 stigma should be implemented with support from national funding for COVID-19 and its health consequences.

## Data availability statement

Deidentified aggregated data may be made available upon request to the corresponding author. Requests to access the datasets should be directed to ED, edrum@hs.uci.edu.

## Ethics statement

This study was certified as exempt from ethical approval using a self-determination form provided by the university’s Institutional Review Board (IRB).

## Author contributions

BB-A, NR, MR, and ED contributed to the design and implementation of the survey. XD, SG, and JW contributed to the design of the statistical analyses and interpretation of the analyses. NR, MR, ED, DG, XD, and SG contributed to manuscript development. BB-A, MR, NR, JW, ED, XD, SG, DG, BG, and JR contributed to the revision of the manuscript for intellectual content. ED agreed to be accountable for all aspects of the work to ensure that questions related to the accuracy or integrity of the work are appropriately investigated and resolved, as corresponding author. All authors contributed to the article and approved the submitted version.

## Conflict of interest

The authors declare that the research was conducted in the absence of any commercial or financial relationships that could be construed as a potential conflict of interest.

## Publisher’s note

All claims expressed in this article are solely those of the authors and do not necessarily represent those of their affiliated organizations, or those of the publisher, the editors and the reviewers. Any product that may be evaluated in this article, or claim that may be made by its manufacturer, is not guaranteed or endorsed by the publisher.
