# Supplementary material for: Anti-Asian racism related stigma, racial discrimination, and protective factors against stigma: a repeated cross-sectional survey among university students during the COVID-19 pandemic
Source: Front Public Health. 2023 Sep 13;11:958932. doi: 10.3389/fpubh.2023.958932 (PMC10524265; doi:10.3389/fpubh.2023.958932)

**Supplementary Materials**

| **Supplementary Table S1**. Chi-square values comparing characteristics with and without missing race | | | | | | | |
| --- | --- | --- | --- | --- | --- | --- | --- |
| **Variables** | **Categories** | **Wave III** | | | **Wave IV** | | |
|  |  | **N** | **(%)** | **Chi-square** | **N** | **(%)** | **Chi-Square** |
| Gender | Male | 63 | (14.82%) | 1.24 | 24 | (6.69%) | 1.34 |
|  | Female | 161 | (17.24%) |  | 42 | (5.02%) |  |
| Education | Undergraduate | 154 | (15.28%) | 3.31 | 45 | (5.40%) | 0.09 |
|  | Graduate | 67 | (19.48%) |  | 21 | (5.83%) |  |
| Age | - | 214 | (16.19%) | 47.69 | 62 | (5.23%) | 29.19 |
| Enacted Stigma | Yes | 37 | (21.89%) | 4.11* | 4 | (3.31%) | 1.26 |
|  | No | 187 | (15.71%) |  | 62 | (5.77%) |  |
| Anticipated Stigma | Yes | 92 | (24.21%) | 22.88** | 8 | (4.12%) | 0.86 |
|  | No | 132 | (13.48%) |  | 58 | (5.79%) |  |
| Internalized Stigma | Yes | 34 | (17.26%) | 0.10 | 8 | (5.23%) | 0.03 |
|  | No | 190 | (16.35%) |  | 58 | (5.56%) |  |
| * p < .05, ** p < .01; counts and percentages are for observations with missing race/ethnicity; gender: 1 = female; 0 = male and other gender identities; education: 1 = graduate; 0 = undergraduate; Age is continuous; Stigma indicators were coded as binary variables (1 = participants selected most of the time, several times, or once/twice; 0 = never). | | | | | | | |

| **Supplementary Table S2**. VIF results of Model 4-5 by waves | | |
| --- | --- | --- |
|  | **Variance Inflation** | |
| **Variables** | **Wave III** | **Wave IV** |
| **Outcome: Anticipated Stigma** |  |  |
| Enacted Stigma | 1.10 | 1.13 |
| Demographic Factors: |  |  |
| Age | 1.79 | 1.81 |
| Gender | 1.12 | 1.17 |
| Race: Asian and Pacific Islander (AAPI) | 2.35 | 2.09 |
| Race: Hispanic or Latinx | 1.93 | 1.83 |
| Race: Non-Hispanic Others | 1.16 | 1.23 |
| Race: Missing | 1.80 | 1.26 |
| Race: Non-Hispanic White | Reference | Reference |
| Socio-economic Factors |  |  |
| Education | 1.79 | 1.91 |
| Residence | 1.05 | 1.06 |
| Employment | 1.14 | 1.16 |
| Social Environmental Factor: People Around |  |  |
| Live Alone | 1.65 | 1.51 |
| Live with Roommates or Neighbors | 1.78 | 1.67 |
| Live with Friends | 1.45 | 1.64 |
| Live with Family | 2.80 | 2.65 |
| Source of COVID-19 Related Information |  |  |
| University Sources | 1.11 | 1.10 |
| Social Media | 1.22 | 1.16 |
| Governmental Sources | 1.11 | 1.16 |
| News | 1.08 | 1.11 |
| Family and Friends | 1.22 | 1.16 |
| **Protective Behaviors: Daily Activities** |  |  |
| Limiting your news consumption to sources considered reliable (meaning with accurate and timely public health information regarding COVID-19). | 1.13 | 1.22 |
| Balancing time between reading news/social media with activities not related to quarantine or isolation (i.e. reading, listening to music). | 1.30 | 1.23 |
| Creating and following a daily routine (including work, exercise, learning, and play). | 1.36 | 1.33 |
| Staying virtually connected with others (using phone calls, text messages, video chat and social media) and discuss your experience and emotions. | 1.15 | 1.15 |
| Relying on pets for emotional support. | 1.15 | 1.14 |
| Maintaining a healthy lifestyle: getting enough sleep, eating well, exercising, avoiding excessive alcohol or drugs. | 1.39 | 1.27 |
| Using telehealth options (phone-based or online) for therapy. | 1.14 | 1.18 |
| Using psychological strategies to manage stress and stay positive (trying not to catastrophize; focusing on what you can do and accepting things you cannot change). | 1.35 | 1.24 |
| Using mindfulness and relaxation exercises (deep breathing, positive imagery, muscle relaxation), including through smartphone applications. | 1.27 | 1.29 |
| Focusing on altruistic reasons for social distancing, quarantine, isolation—to reduce transmission of COVID-19 and protect the most vulnerable. | 1.24 | 1.17 |
| Volunteering to help others who are most vulnerable. | 1.16 | 1.19 |
| **Stigma Addressing Activities** |  |  |
| Sharing what you learn about preventing disease with your family and friends. | 1.18 | 1.43 |
| Speaking out against negative behaviors, including negative statements on social media about groups of people, or exclusion of people who pose no risk from regular activities. | 1.39 | 1.45 |
| Being cautious about the images that you see and share in media and online. Making sure they do not reinforce stereotypes. | 1.28 | 1.42 |
| Providing social support for people who have returned from affected areas or are worried about friends or family in the affected areas. | 1.31 | 1.38 |
| **Stigma Resistance Activities** |  |  |
| Resisting stigma means speaking up when others say negative things about Asians or Asian Americans regarding COVID-19. | 1.50 | 1.79 |
| I help others resist stigma by showing I believe in them. | 1.54 | 1.67 |
| I help others see they should not be ashamed about being Asian or Asian American. | 1.53 | 1.62 |
| To resist stigma, I think positive things about myself. | 1.35 | 1.31 |
| When I encounter stigma, I can think of why these attitudes are wrong. | 1.43 | 1.68 |
| **Outcome: Internalized Stigma** |  |  |
| Enacted Stigma | 1.37 | 1.62 |
| Anticipated Stigma | 1.49 | 1.63 |
| Demographic Factors |  |  |
| Age | 1.79 | 1.81 |
| Gender | 1.12 | 1.17 |
| Race: Asian and Pacific Islander (AAPI) | 2.47 | 2.15 |
| Race: Hispanic or Latinx | 1.93 | 1.83 |
| Race: Non-Hispanic Others | 1.16 | 1.24 |
| Race: Non-Hispanic White | 1.86 | 1.26 |
| Race: Missing | Reference | Reference |
| Socio-economic Factors |  |  |
| Education | 1.79 | 1.91 |
| Residence | 1.05 | 1.06 |
| Employment | 1.15 | 1.16 |
| Social Environmental Factor: People Around |  |  |
| Live Alone | 1.65 | 1.51 |
| Live with Roommates or Neighbors | 1.78 | 1.67 |
| Live with Friends | 1.45 | 1.64 |
| Live with Family | 2.80 | 2.65 |
| Source of COVID-19 Related Information |  |  |
| University Sources | 1.11 | 1.10 |
| Social Media | 1.22 | 1.17 |
| Governmental Sources | 1.11 | 1.16 |
| News | 1.08 | 1.10 |
| Family and Friends | 1.23 | 1.16 |
| **Protective Behaviors: Daily Activities** |  |  |
| Limiting your news consumption to sources considered reliable (meaning with accurate and timely public health information regarding COVID-19). | 1.13 | 1.23 |
| Balancing time between reading news/social media with activities not related to quarantine or isolation (i.e. reading, listening to music). | 1.30 | 1.23 |
| Creating and following a daily routine (including work, exercise, learning, and play). | 1.36 | 1.33 |
| Staying virtually connected with others (using phone calls, text messages, video chat and social media) and discuss your experience and emotions. | 1.15 | 1.15 |
| Relying on pets for emotional support. | 1.15 | 1.14 |
| Maintaining a healthy lifestyle: getting enough sleep, eating well, exercising, avoiding excessive alcohol or drugs. | 1.39 | 1.28 |
| Using telehealth options (phone-based or online) for therapy. | 1.14 | 1.19 |
| Using psychological strategies to manage stress and stay positive (trying not to catastrophize; focusing on what you can do and accepting things you cannot change). | 1.35 | 1.24 |
| Using mindfulness and relaxation exercises (deep breathing, positive imagery, muscle relaxation), including through smartphone applications. | 1.27 | 1.29 |
| Focusing on altruistic reasons for social distancing, quarantine, isolation—to reduce transmission of COVID-19 and protect the most vulnerable. | 1.24 | 1.17 |
| Volunteering to help others who are most vulnerable. | 1.16 | 1.19 |
| **Stigma Addressing Activities** |  |  |
| Sharing what you learn about preventing disease with your family and friends. | 1.18 | 1.43 |
| Speaking out against negative behaviors, including negative statements on social media about groups of people, or exclusion of people who pose no risk from regular activities. | 1.39 | 1.45 |
| Being cautious about the images that you see and share in media and online. Making sure they do not reinforce stereotypes. | 1.28 | 1.42 |
| Providing social support for people who have returned from affected areas or are worried about friends or family in the affected areas. | 1.31 | 1.38 |
| **Stigma Resistance Activities** |  |  |
| Resisting stigma means speaking up when others say negative things about Asians or Asian Americans regarding COVID-19. | 1.51 | 1.79 |
| I help others resist stigma by showing I believe in them. | 1.55 | 1.67 |
| I help others see they should not be ashamed about being Asian or Asian American. | 1.53 | 1.62 |
| To resist stigma, I think positive things about myself. | 1.36 | 1.31 |
| When I encounter stigma, I can think of why these attitudes are wrong. | 1.43 | 1.68 |
| ** p < .05, ** p < .01* | | |

| **Supplementary Table S3.** Logistic regression results of sociodemographic, social environmental, and behavioral factors associated with anticipated stigma by wave | | | | |
| --- | --- | --- | --- | --- |
|  | **Wave III** | | **Wave IV** | |
| **Variables** | **aOR** | **95% CI** | **aOR** | **95% CI** |
| Enacted Stigma | 38.66** | (18.05, 82.79) | 51.88** | (24.21, 111.18) |
| **Demographic Factors** |  |  |  |  |
| Age | 1.00 | (0.93, 1.08) | 1.04 | (0.96, 1.12) |
| Gender | 1.08 | (0.70, 1.66) | 0.79 | (0.44, 1.41) |
| Race: Asian and Pacific Islander (AAPI) | 7.50** | (3.72, 15.15) | 7.06** | (2.79, 17.91) |
| Race: Hispanic or Latinx | 0.75 | (0.31, 1.79) | 1.65 | (0.58, 4.68) |
| Race: Non-Hispanic Others | 2.58 | (0.58, 11.48) | 12.98* | (2.43, 69.39) |
| Race: Missing | 7.68** | (3.55, 16.63) | 1.48 | (0.27, 8.17) |
| Race: Non-Hispanic White | Reference | Reference | Reference | Reference |
| **Socio-economic Factors** |  |  |  |  |
| Education | 0.91 | (0.44, 1.88) | 1.59 | (0.68, 3.70) |
| Residence | 0.72 | (0.36, 1.43) | 0.90 | (0.36, 2.28) |
| Employment | 1.25 | (0.82, 1.89) | 1.00 | (0.57, 1.73) |
| **Social Environmental Factors: People Around** |  |  |  |  |
| Live Alone | 1.25 | (0.37, 4.29) | 0.97 | (0.14, 6.52) |
| Live with Roommates or Neighbors | 1.27 | (0.54, 2.97) | 1.21 | (0.44, 3.27) |
| Live with Friends | 1.18 | (0.48, 2.89) | 1.35 | (0.45, 4.10) |
| Live with Family | 1.21 | (0.51, 2.86) | 1.26 | (0.47, 3.33) |
| **Source of COVID-19 Related Information** |  |  |  |  |
| University Sources | 1.23 | (0.80, 1.89) | 1.33 | (0.73, 2.41) |
| Social Media | 1.46 | (0.95, 2.24) | 0.75 | (0.43, 1.31) |
| Governmental Sources | 0.85 | (0.56, 1.29) | 0.58 | (0.32, 1.03) |
| News | 0.96 | (0.59, 1.58) | 0.90 | (0.49, 1.66) |
| Family and Friends | 1.28 | (0.83, 1.99) | 0.64 | (0.37, 1.10) |
| **Protective Behaviors: Daily Activities** | | | | |
| Limiting your news consumption to sources considered reliable (meaning with accurate and timely public health information regarding COVID-19). | 1.00 | (0.53, 1.88) | 3.59* | (1.09, 11.83) |
| Balancing time between reading news/social media with activities not related to quarantine or isolation (i.e. reading, listening to music). | 0.79 | (0.38, 1.64) | 0.52 | (0.20, 1.37) |
| Creating and following a daily routine (including work, exercise, learning, and play). | 0.72 | (0.42, 1.23) | 0.93 | (0.46, 1.88) |
| Staying virtually connected with others (using phone calls, text messages, video chat and social media) and discuss your experience and emotions. | 0.46 | (0.19, 1.10) | 1.61 | (0.45, 5.72) |
| Relying on pets for emotional support. | 1.03 | (0.70, 1.53) | 1.04 | (0.61, 1.77) |
| Maintaining a healthy lifestyle: getting enough sleep, eating well, exercising, avoiding excessive alcohol or drugs. | 0.92 | (0.49, 1.70) | 0.27* | (0.13, 0.59) |
| Using telehealth options (phone-based or online) for therapy. | 0.96 | (0.58, 1.59) | 0.65 | (0.34, 1.26) |
| Using psychological strategies to manage stress and stay positive (trying not to catastrophize; focusing on what you can do and accepting things you cannot change). | 1.00 | (0.58, 1.71) | 1.56 | (0.77, 3.18) |
| Using mindfulness and relaxation exercises (deep breathing, positive imagery, muscle relaxation), including through smartphone applications. | 1.13 | (0.75, 1.70) | 0.90 | (0.50, 1.60) |
| Focusing on altruistic reasons for social distancing, quarantine, isolation—to reduce transmission of COVID-19 and protect the most vulnerable. | 1.34 | (0.63, 2.88) | 0.95 | (0.37, 2.42) |
| Volunteering to help others who are most vulnerable. | 1.49 | (0.98, 2.24) | 1.21 | (0.70, 2.08) |
| **Stigma Addressing Activities** |  |  |  |  |
| Sharing what you learn about preventing disease with your family and friends. | 0.88 | (0.47, 1.65) | 1.75 | (0.87, 3.51) |
| Speaking out against negative behaviors, including negative statements on social media about groups of people, or exclusion of people who pose no risk from regular activities. | 0.96 | (0.63, 1.48) | 1.05 | (0.58, 1.92) |
| Being cautious about the images that you see and share in media and online. Making sure they do not reinforce stereotypes. | 1.04 | (0.57, 1.88) | 1.10 | (0.56, 2.19) |
| Providing social support for people who have returned from affected areas or are worried about friends or family in the affected areas. | 0.97 | (0.64, 1.48) | 1.42 | (0.79, 2.56) |
| **Stigma Resistance Activities** |  |  |  |  |
| Resisting stigma means speaking up when others say negative things about Asians or Asian Americans regarding COVID-19. | 2.38* | (1.12, 5.03) | 0.51 | (0.20, 1.31) |
| I help others resist stigma by showing I believe in them. | 0.78 | (0.47, 1.28) | 1.32 | (0.59, 2.93) |
| I help others see they should not be ashamed about being Asian or Asian American. | 0.97 | (0.59, 1.60) | 1.67 | (0.77, 3.61) |
| To resist stigma, I think positive things about myself. | 1.32 | (0.84, 2.08) | 0.70 | (0.39, 1.24) |
| When I encounter stigma, I can think of why these attitudes are wrong. | 0.86 | (0.41, 1.83) | 1.17 | (0.44, 3.07) |
| ** p < .05, ** p < .01; gender: 1 = female; 0 = male and other gender identities (reference); education: 1 = graduate; 0 = undergraduate (reference); Age is continuous; Residence: 1 = urban; 0 = rural (reference); employment: 1 = employed (full-time or part-time); 0 = unemployed (reference); Stigma indicators were coded as binary variables (1 = participants selected most of the time, several times, or once/twice; 0 = never); Social Environmental Factor: 1 = yes; 0 = no (reference); source of COVID-19 related information: 1 = yes; 0 = no (reference); protective behaviors daily activities: 1 = always or sometimes; 0 = never (reference); protective behaviors stigma addressing activities: 1 = yes; 0 = no or don't remember (reference); protective behaviors stigma resistance activities: 1 = strongly agree, agree; 0 = strongly disagree, disagree, and neither agree/disagree (reference).* | | | | |
|  |  |  |  |  |
|  |  |  |  |  |
|  |  |  |  |  |
|  |  |  |  |  |
|  |  |  |  |  |

| **Supplementary Table S4.** Logistic regression results of Sociodemographic, social environmental, and behavioral factors associated with internalized stigma by wave | | | | |
| --- | --- | --- | --- | --- |
|  | Wave III | | Wave IV | |
| **Variables** | aOR | 95% CI | aOR | 95% CI |
| Enacted Stigma | 3.39** | (1.87, 6.14) | 4.98** | (2.44, 10.19) |
| Anticipated Stigma | 2.72** | (1.59, 4.64) | 3.33** | (1.72, 6.47) |
| **Demographic Factors** |  |  |  |  |
| Age | 1.03 | (0.96, 1.10) | 1.01 | (0.94, 1.09) |
| Gender | 1.13 | (0.68, 1.88) | 1.35 | (0.73, 2.50) |
| Race: Asian and Pacific Islander (AAPI) | 0.76 | (0.38, 1.52) | 1.84 | (0.77, 4.37) |
| Race: Hispanic or Latinx | 0.74 | (0.35, 1.57) | 1.58 | (0.61, 4.09) |
| Race: Non-Hispanic Others | 1.25 | (0.27, 5.88) | 2.44 | (0.50, 11.97) |
| Race: Non-Hispanic White | 0.83 | (0.37, 1.83) | 2.08 | (0.51, 8.54) |
| Race: Missing | Reference | Reference | Reference | Reference |
| **Socio-economic Factors** |  |  |  |  |
| Education | 0.37* | (0.15, 0.91) | 0.76 | (0.30, 1.93) |
| Residence | 0.74 | (0.34, 1.61) | 1.03 | (0.41, 2.56) |
| Employment | 0.89 | (0.55, 1.43) | 1.20 | (0.69, 2.09) |
| **Social Environmental Factors: People Around** |  |  |  |  |
| Live Alone | 2.38 | (0.54, 10.36) | 0.50 | (0.08, 3.16) |
| Live with Roommates or Neighbors | 0.76 | (0.29, 2.01) | 0.25* | (0.07, 0.83) |
| Live with Friends | 2.96* | (1.10, 7.97) | 0.60 | (0.18, 1.96) |
| Live with Family | 1.58 | (0.58, 4.31) | 0.46 | (0.17, 1.28) |
| **Source of COVID-19 Related Information** |  |  |  |  |
| University Sources | 1.35 | (0.81, 2.24) | 1.37 | (0.74, 2.54) |
| Social Media | 0.96 | (0.59, 1.57) | 1.03 | (0.59, 1.80) |
| Governmental Sources | 1.24 | (0.73, 2.08) | 0.86 | (0.47, 1.57) |
| News | 0.66 | (0.38, 1.12) | 1.48 | (0.76, 2.88) |
| Family and Friends | 0.88 | (0.53, 1.44) | 1.59 | (0.88, 2.85) |
| **Protective Behaviors: Daily Activities** | | | | |
| Limiting your news consumption to sources considered reliable (meaning with accurate and timely public health information regarding COVID-19). | 0.56 | (0.30, 1.05) | 1.02 | (0.38, 2.75) |
| Balancing time between reading news/social media with activities not related to quarantine or isolation (i.e. reading, listening to music). | 0.84 | (0.38, 1.83) | 1.36 | (0.48, 3.87) |
| Creating and following a daily routine (including work, exercise, learning, and play). | 0.78 | (0.43, 1.40) | 1.08 | (0.53, 2.21) |
| Staying virtually connected with others (using phone calls, text messages, video chat and social media) and discuss your experience and emotions. | 0.76 | (0.32, 1.81) | 0.52 | (0.16, 1.70) |
| Relying on pets for emotional support. | 1.39 | (0.88, 2.19) | 1.29 | (0.75, 2.23) |
| Maintaining a healthy lifestyle: getting enough sleep, eating well, exercising, avoiding excessive alcohol or drugs. | 0.58 | (0.31, 1.11) | 0.68 | (0.30, 1.54) |
| Using telehealth options (phone-based or online) for therapy. | 1.14 | (0.64, 2.05) | 1.93* | (1.05, 3.55) |
| Using psychological strategies to manage stress and stay positive (trying not to catastrophize; focusing on what you can do and accepting things you cannot change). | 0.83 | (0.46, 1.48) | 1.09 | (0.54, 2.21) |
| Using mindfulness and relaxation exercises (deep breathing, positive imagery, muscle relaxation), including through smartphone applications. | 1.09 | (0.67, 1.77) | 1.18 | (0.66, 2.11) |
| Focusing on altruistic reasons for social distancing, quarantine, isolation—to reduce transmission of COVID-19 and protect the most vulnerable. | 0.55 | (0.26, 1.13) | 1.39 | (0.50, 3.85) |
| Volunteering to help others who are most vulnerable. | 1.42 | (0.89, 2.29) | 0.89 | (0.51, 1.56) |
| **Stigma Addressing Activities** |  |  |  |  |
| Sharing what you learn about preventing disease with your family and friends | 1.54 | (0.68, 3.49) | 0.71 | (0.36, 1.41) |
| Speaking out against negative behaviors, including negative statements on social media about groups of people, or exclusion of people who pose no risk from regular activities. | 0.95 | (0.57, 1.56) | 0.79 | (0.42, 1.45) |
| Being cautious about the images that you see and share in media and online. Making sure they do not reinforce stereotypes. | 1.25 | (0.59, 2.63) | 1.31 | (0.65, 2.68) |
| Providing social support for people who have returned from affected areas or are worried about friends or family in the affected areas. | 1.34 | (0.81, 2.23) | 1.54 | (0.85, 2.81) |
| **Stigma Resistance Activities** |  |  |  |  |
| Resisting stigma means speaking up when others say negative things about Asians or Asian Americans regarding COVID-19. | 1.64 | (0.71, 3.79) | 0.42 | (0.16, 1.09) |
| I help others resist stigma by showing I believe in them. | 0.74 | (0.42, 1.33) | 1.80 | (0.77, 4.21) |
| I help others see they should not be ashamed about being Asian or Asian American. | 1.09 | (0.61, 1.94) | 1.30 | (0.62, 2.73) |
| To resist stigma, I think positive things about myself. | 0.64 | (0.39, 1.06) | 0.48* | (0.27, 0.85) |
| When I encounter stigma, I can think of why these attitudes are wrong. | 1.11 | (0.51, 2.46) | 0.85 | (0.36, 2.01) |
| **p< .05, **p<.01; gender: 1=female; 0=male and other gender identities (reference); education: 1= graduate; 0=undergraduate (reference); Age is continuous; Residence: 1=urban. 0=rural (reference); employment: 1=employed (full-time or part-time); 0=unemployed (reference); Stigma indicators were coded as binary variables (1=participants selected most of the time, several times, or once/twice; 0=never); Social environment: 1=yes; 0=no (reference); source of COVID-19 related information: 1=yes, 0=no (reference); protective behaviors daily activities: 1=always or sometimes, 0=never (reference); stigma addressing activities: 1=yes, 0= no or don’t remember (reference); stigma resistance activities: 1=strongly agree, agree, 0= strongly agree, disagree, and neither aggree/disagree (reference).* | | | | |

**Supplementary Figure S1.** Number of respondents in Waves III and IV


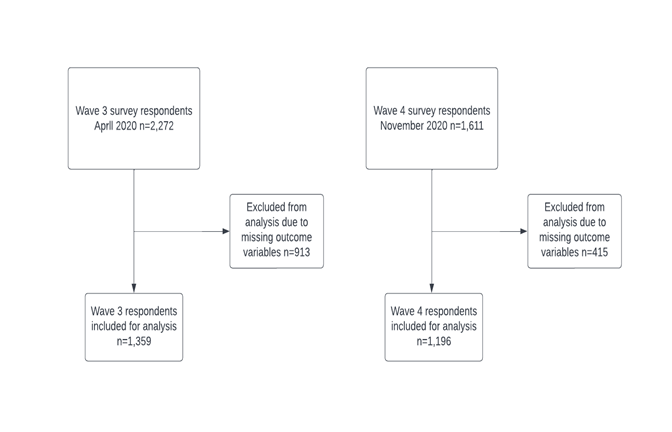


**Supplementary Figure S2.** Directed Acyclic Graph **(**DAG) of race and enacted stigma


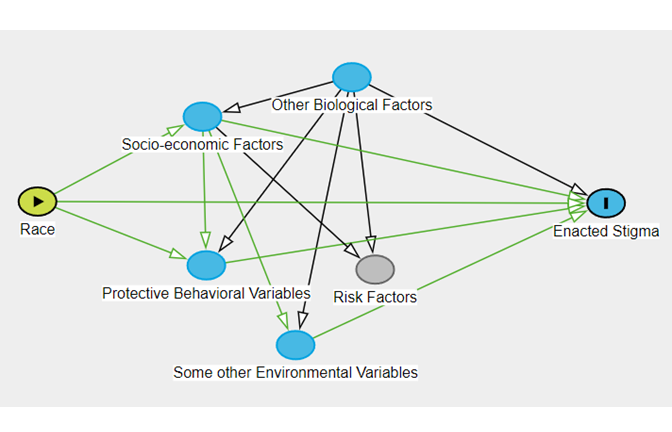


**Supplementary Figure S3.** DAG of race and anticipated stigma


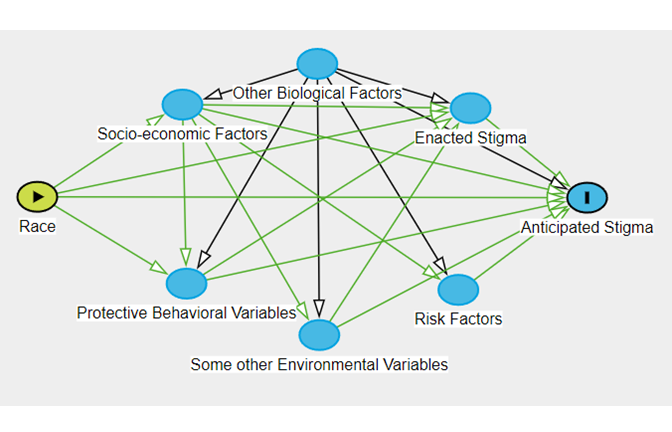


**Supplementary Figure S4.** DAG of race and internalized stigma


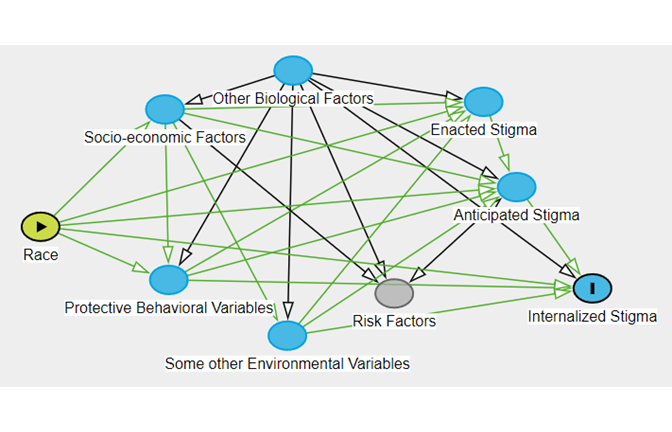


**Supplementary Figure S5.** Potential risk and protective factors for the odds of anticipated stigma
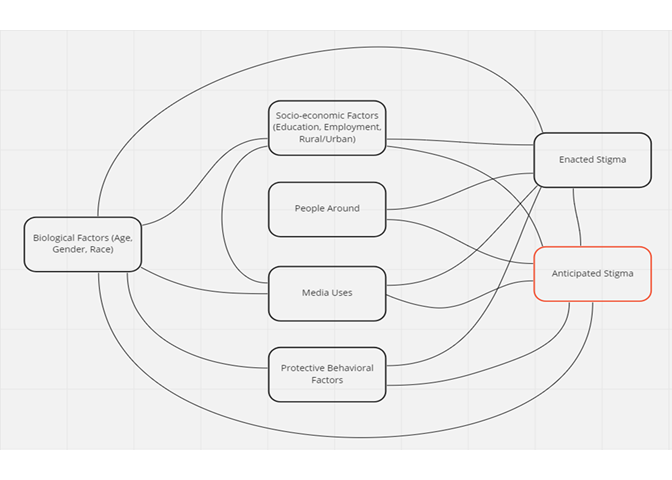


**Supplementary Figure S6.** Potential risk and protective factors for the odds of internalized stigma


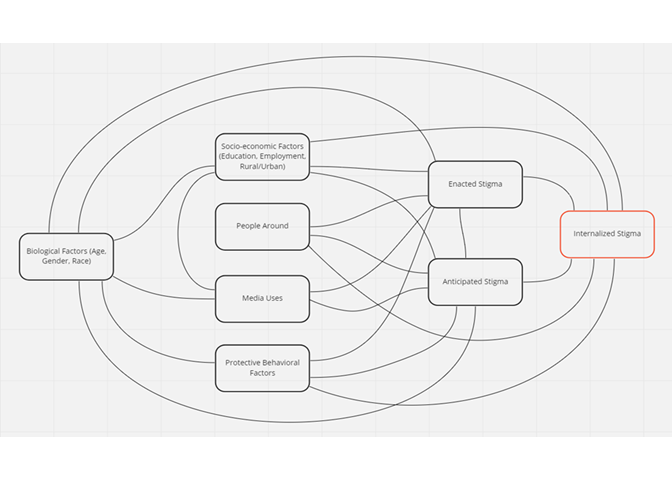

Supplement: Supplementary file 1 [file Table_1.docx]
