# Supplementary figures and images for: Anti-Asian racism related stigma, racial discrimination, and protective factors against stigma: a repeated cross-sectional survey among university students during the COVID-19 pandemic
Source: Front Public Health. 2023 Sep 13;11:958932. doi: 10.3389/fpubh.2023.958932 (PMC10524265; doi:10.3389/fpubh.2023.958932)

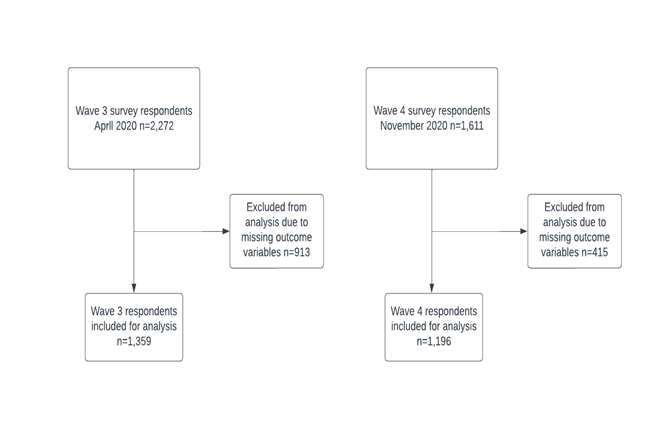

Supplement: Supplementary file 2 [file Image_1.TIF]

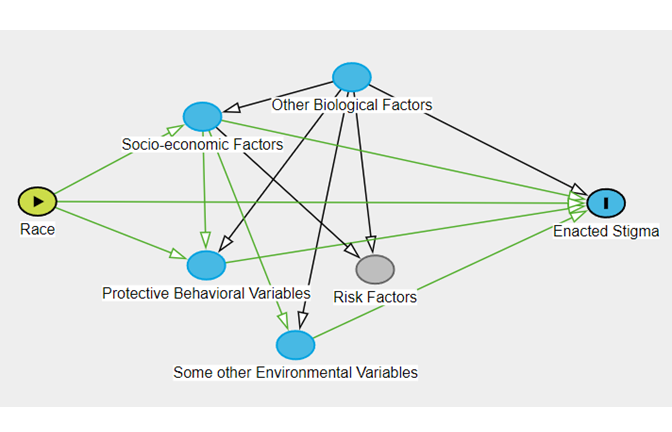

Supplement: Supplementary file 3 [file Image_2.TIF]

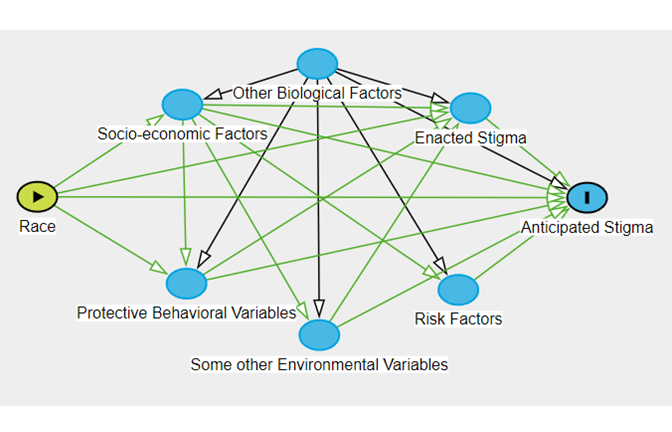

Supplement: Supplementary file 4 [file Image_3.TIF]

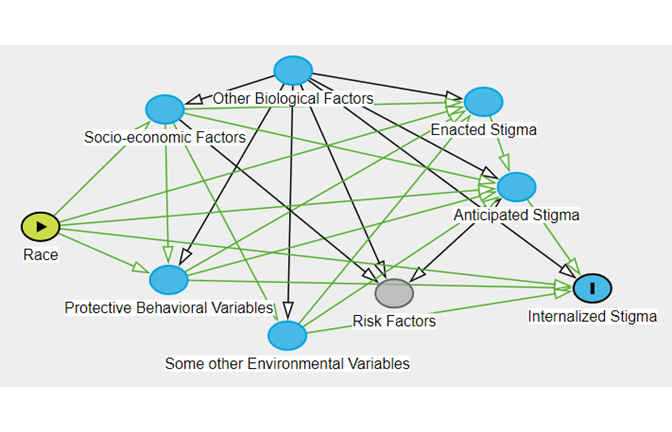

Supplement: Supplementary file 5 [file Image_4.TIF]

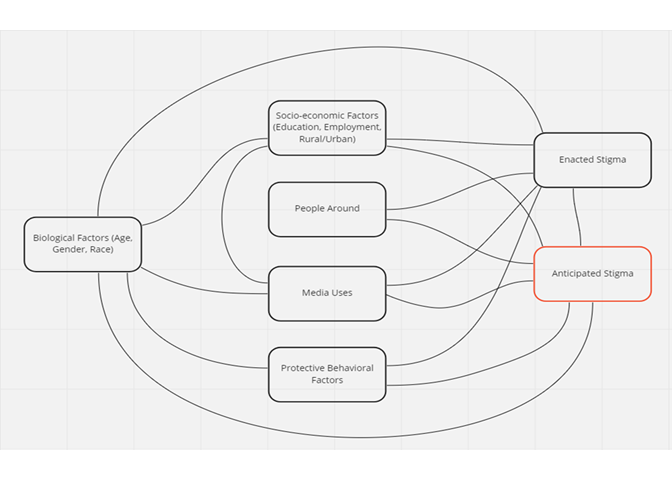

Supplement: Supplementary file 6 [file Image_5.TIF]

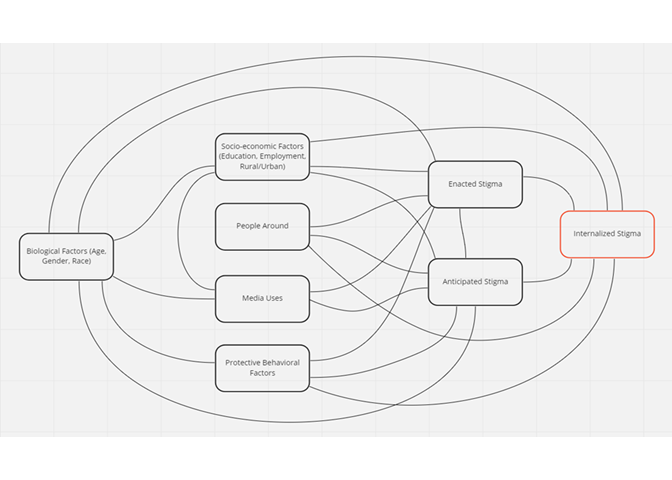

Supplement: Supplementary file 7 [file Image_6.TIF]
